# Comparative diagnostic accuracy of the IOTA SRR and LR2 scoring systems for discriminating between malignant and Benign Adnexal masses by junior physicians in Chinese patients: a retrospective observational study

**DOI:** 10.1186/s12905-023-02719-z

**Published:** 2023-11-08

**Authors:** Cai Tian, Shu-Bin Wen, Cong-Ying Zhao, Xiao-Nan Yan, Jie-Xian Du

**Affiliations:** Department of gynecology, The second Hospital of Hebei Medical University, NO.215 of He ping West Road, Xinhua District, Shijiazhuang, 050000 China

**Keywords:** Ovarian cancer, Adnexal tumours, Contrast-enhanced ultrasound, Ultrasound diagnosis, IOTA, SRR, LR2

## Abstract

**Background:**

The accuracy of ultrasound in distinguishing benign from malignant adnexal masses is highly correlated with the experience of ultrasound physicians. In China, most of ultrasound differentiation is done by junior physicians.

**Purpose:**

To compare the diagnostic performance of the International Ovarian Tumour Analysis (IOTA) Simple Rules Risk (SRR) and IOTA Logistic Regression Model 2 (LR2) scoring systems in Chinese patients with adnexal masses.

**Methods:**

Retrospective analysis of ovarian cancer tumor patients who underwent surgery at a hospital in China from January 2016 to December 2021. Screening patients with at least one adnexal mass on inclusion and exclusion criteria. Two trained junior physicians evaluated each mass using the two scoring systems. A receiver operating characteristic curve was used to test the diagnostic performance of each system.

**Results:**

A total of 144 adnexal masses were retrospectively collected. Forty masses were histologically diagnosed as malignant. Compared with premenopausal women, postmenopausal women had a much higher rate of malignant masses. The sensitivity, specificity, positive predictive value (PPV), negative predictive value (NPV) of the SRR was 97.5% (95% CI: 86.8 -99.9%), 82.7% (95% CI: 74.0 -89.4%), 68.4% (95% CI: 58.7 -76.8%) and 98.9% (95% CI: 92.5 -99.8%). The sensitivity, specificity, PPV, NPV of the LR2 were 90.0% (95% CI: 76.5 -97.2%), 89.4% (95% CI: 81.9 -94.6%), 76.6% (95% CI: 65.0 -85.2%), and 95.9% (95% CI: 90.2 -98.3%). There was good agreement between two scoring systems, with 84.03% total agreement and a kappa value of 0.783 (95% CI: 0.70-0.864). The areas under the curve for predicting malignant tumours using SRR and LR2 were similar for all patients (*P* > 0.05 ).

**Conclusion:**

The two scoring systems can effectively distinguish benign from malignant adnexal masses. Both scoring systems have high diagnostic efficacy, and diagnostic efficacy is stable, which can provide an important reference for clinical decision making.

## Introduction

Ovarian cancer (OC), which occurs in ovarian tissue deep within the pelvis, is the third most prevalent type of gynaecological cancer in the world [1]. According to statistics, more than 75% of DCs are diagnosed in the late stage, and in recent years, the improvement of patient treatment plans has steadily increased the overall 5-year survival rate of these patients to approximately 47% [2]. Furthermore, in the most common histological type, i.e. advanced serous carcinoma cases, more than 90% of patients are diagnosed with advanced-stage tumours, and their 5-year survival rate is less than 30% [3]. OC also poses a huge threat to the lives and health of Chinese women. From 1990 to 2019, the age standardized mortality rate of OC increased from 1.76 to 2.88 per 100,000, and the crude mortality rate increased from 1.4 to 4.17 per 100,000 [4].

Early diagnosis is important for reducing mortality from OC; however, there is currently no standardised strategy for the screening or early detection of OC [5].

Imaging techniques, such as ultrasound (US) and magnetic resonance imaging (MRI), have been used in the preoperative assessment of adnexal tumours. Because of its low cost and accessibility, US has proved to be the most useful diagnostic tool for adnexal masses [6]. However, due to the long-standing lack of recognized standardized standards or diagnostic models in gynecological ultrasound examination to derive the nature of lesions, the difficulty of ultrasound diagnosis of adnexal tumors is high, with high rates of missed diagnosis and misdiagnosis [7]. And the accuracy of ultrasound in differentiating between benign and malignant adnexal masses is highly correlated with the experience of the sonographer [8]. To improve the diagnostic accuracy of ultrasound for adnexal masses, the International Ovarian Tumour Analysis (IOTA) group, a European workforce, developed several US-based prognostic models, including Simple Rule (SR), Logistic Regression Model 2 (LR2), Simple Descriptor and Simple Rules Risk (SRR) [8–10].

The SR model describes five typical features of benign (B) tumours and five typical features of malignant (M) tumours. The model is easy to use and can compensate for junior physicians’ lack of experience [6]. External validation of the SR model showed that it had high sensitivity (87.5–95.2%) and specificity (87.6–100%) for discriminating between malignant and benign adnexal tumours [11, 12]. However, SR is a dichotomous model, with approximately 25% of unclassifiable lesions falling into an ‘inconclusive’ category, which has certain limitations in its use [13]. The SRR scoring system conducted a numerical risk assessment of the malignancy of pelvic lesions based on the B and M features of the SR model [13]. Hiett et al. [13] found SRR to have high sensitivity in the preoperative differentiation between malignant and benign pelvic tumours. Czekierdowski et al. [10] asserted that the SRR scoring system could improve the diagnosis of adnexal tumours in pregnancy among less experienced sonographers. The LR2 model is a ovarian tumor benign and malignant prediction model proposed by IOTA, which focuses more on analyzing ultrasound image features [14], and it is one of the most widely used adnexal lesion diagnostic systems in clinical practice [12] and includes only six variables. External validation studies show the sensitivity and specificity of this model to be as high as 0.93 and 0.84, respectively [15]. LR2 has a wider range of applications and is suitable for all ovarian masses. Some scholars claim that it can be used as an auxiliary method for junior physicians to diagnose ovarian tumors [16].

The above prediction models were mainly constructed and validated based on data from European populations. To the best of our knowledge, no studies have been published comparing the accuracy of IOTA SRR and IOTA LR2 in discriminating between benign and malignant adnexal masses in Chinese patients. In China, due to the insufficient number of experts in ultrasound examination, most of the judgments of benign and malignant tumors are completed by inexperienced junior physicians and junior physicians also have difficulty in differential diagnosis of tumors [17]. However, the use of scoring systems such as IOTA SRR and IOTA LR2 may enable these physicians to reduce the impact of subjective judgments and improve their accuracy and consistency. In the clinical diagnosis of ovarian tumor patients, improving the clinical early differential value of tumor pathological types can help provide accurate data support for early treatment of diseases [18]. At the same time, how to minimize diagnostic errors caused by differences in experience and further improve the ultrasound diagnosis rate of accessory tumors has always been the focus of research by Chinese ultrasound physicians. Therefore, the current study aims to compare the diagnostic accuracy of junior physicians using the IOTA SRR and LR2 scoring systems for benign and malignant tumours through a retrospective study. The puepose is to explore the value of SRR and LR2 scoring systems in improving the diagnostic ability of Chinese junior physicians for adnexal masses, providing powerful tools for early intervention and treatment of patients, and exploring better methods for determining the benign and malignant nature of adnexal masses in clinical.

## Methods

### Patients

The current study retrospectively analyzed the basic information, hospitalization information, and ultrasound images of ovarian cancer tumor patients who underwent surgery in a hospital in China from January 2016 to December 2021 and were diagnosed through postoperative pathological examination. The current study belongs to convenient sampling.

According to the Helsinki Declaration, this retrospective observational study was approved by the Ethics Committee of our hospital. All participants have informed written consent to publish the data. Therefore, there is no sensitive data in the current study.

The inclusion criteria were as follows: (1) patients diagnosed by US with at least one adnexal mass; (2) patients over the age of 18; (3) patients whose lesions had been removed surgically and evaluated pathologically; and (4) preoperative ultrasound examination was performed according to the requirement and the information of ultrasound image was preserved completely. The exclusion criteria were as follows: (1) pregnant women with adnexal masses; and (2) patients with a history of a malignant gynaecological tumour prior to the diagnosis of an adnexal mass. The study ultimately included a total of 128 patients with 144 adnexal masses.

### Ultrasound evaluation

In all cases, transvaginal US examination was the primary scanning modality. A transabdominal US was performed only when the mass was too large to be observed by transvaginal US. A Voluson E8 (GE Healthcare, Milwaukee, WI, USA) US machine was used. The intracavitary transducer frequency was set to 5.0–9.0 MHz, and the abdominal probe frequency was 3.5–5.0 MHz. According to previous similar studies by Chinese scholars [6], the US characteristics of each mass were described using IOTA terminology [19].

The IOTA SRR scoring system provided a numeric risk estimate of the malignancy of pelvic lesions based on the B- and M-features of the SR model [20]. The B-features included the following: B1, unilocularity; B2, the presence of solid areas of a diameter no greater than 7 mm; B3, the presence of acoustic shadows; B4, the presence of smooth, multilocular masses of a diameter less than 100 mm; and B5, the absence of intratumoural blood flow (colour score: 1). The M-features included the following: M1, the presence of irregular and solid masses; M2, the presence of ascites; M3, the presence of at least four papillary structures; M4, the presence of irregular, multilocular solid areas of a diameter greater than 100 mm; and M5, strong blood flow (colour score: 4) [10]. The presence or absence of each B- and M-feature was entered into the SRR calculator (https://homes.esat.kuleuven.be/~sistawww/biomed/ssrisk/). Following previous studies, the current study used two classifications of SRR risk, i.e. low (≤ 20%) and high (> 20%) [10].

Next, the IOTA LR2 scores were calculated based on the following six features: (a) patient age; (b) presence of ascites; (c) solid papillary blood flow; (d) maximal solid component diameter; (e) irregularity of internal cyst walls; and (f) the presence of acoustic shadows [12]. The LR2 formula used to determine the probability of malignancy was *y* = 1 / (1 + exp (˗ *z*)), where *z* = ˗5.3718 + 0.0354 (a) + 1.6159 (b) + 1.1768 (c) + 0.0697 (d) + 0.9586 (e) ˗ 2.9486 (f). A probability greater than 10% was considered to represent a high risk of malignancy [12].

Two junior physicians with US diagnostic experience < 2 years analysed the images and evaluated each mass using the SRR and LR2 systems independently. Where results differed, the two physicians worked together with a senior doctor with > 10 years of US diagnostic experience to analyse the images. Prior to the start of the study, two junior physicians were trained in the theoretical and practical aspects of the SRR and LR2 models. The specific content included standardized training for ovarian benign tumors, standardized training for ovarian malignant tumors, and IOTA diagnostic model training. This training was conducted by senior professors in the form of a seminar, where the professor first imparts knowledge and then discusses with the physician. Each training lasts for 2.5 to 3 h and was conducted three times. The entire training was completed within half a month. After the training is completed, two physicians used SRR and LR2 models to diagnose 40 randomly selected adnexal masses, and performed inter group consistency analysis on their diagnostic results. The results showed that the kappa(k) value of the SRR and LR2 models were 0.92 (95% confidence interval [CI]: 0.84, 1.00) and 0.87 (95% CI: 0.78, 1.00), respectively, with good consistency results. The results of good consistency can increase the reliability and reliability of the study, and provide support for the validity of the study.

Both physicians did not participate in patient information and image collection, and both physicians followed the double-blind principle in the image evaluation process.

### Data collection

Researchers retrospectively collected basic and clinical information of each patient including age, menopausal status and cancer antigen 125 (CA125) levels using the patient’s medical record homepage and hospital information systems. Experienced physicians used clear histological diagnosis as the gold standard for diagnosing each resected mass.

### Quality control

(1) Strictly screen research subjects based on inclusion and exclusion criteria to avoid selection bias; (2) Provide a clear data collection process to ensure the accuracy and completeness of research data; (3) Two junior physicians were trained to ensure that they are familiar with and correctly use the SRR and LR2 scoring systems; (4) After the training, a portion of the samples were randomly selected for simulation, and consistency evaluation was conducted on two junior physicians to ensure the reliability of the study.

### Statistical analysis

Data were analysed using the SPSS v.22.0 and MedCalc v.19.0.5 statistical software. Categorical data were described as numbers and percentages and compared using chi-squared (χ^2^) or Fisher’s exact tests. Quantitative data were described as mean ± standard deviation and compared using a *t*-test. Receiver operating characteristic (ROC) curves were used to evaluate the diagnostic accuracy of the two scoring systems in terms of discriminating between benign and malignant adnexal masses. The ROC curves reflect the relationship between the sensitivity and specificity of the curve [21]. The x-axis is 1-specificity, also known as the false positive rate, and the closer the x-axis is to zero, the higher the accuracy; the y-axis reflects sensitivity and is also known as the true positive rate (sensitivity); the larger the y-axis, the better the accuracy. Based on the position of the curve, the entire graph was divided into two parts [22]. The area under the curve (AUC) is used to indicate the accuracy of the prediction. The higher the AUC, the greater the AUC and the higher the prediction accuracy. The closer the curve is to the upper left corner (the smaller the x, the larger the y), the higher the prediction accuracy. DeLong’s test was used to compare the AUCs of the ROC curves. Agreement by κ-values was considered poor at 0–0.20, fair at 0.21–0.40, moderate at 0.41–0.60, good at 0.61–0.80 and very good at 0.81–1.00. The results were considered statistically significant at *p* < 0.05.

## Results

### Patient clinical and pathological results

A total of 128 patients who met the inclusion criteria were included in the current study. The mean age of the enrolled patients was 41.19 ± 16.97 years. Seventy-four patients (57.81%) were premenopausal, with a mean age of 28.95 ± 9.51 years, and 54 patients (42.19%) were postmenopausal, with a mean age of 57.96 ± 8.36 years. Sixteen patients (12.50%) had bilateral lesions and 45 (35.16%) patients had increased CA125 levels, including 31 (57.41%) postmenopausal women and 14 (18.92%) premenopausal women. The clinical characteristics of the enrolled patients are summarised in Table 1.

**Table 1:** The clinical characteristics of the 128 patients

Characteristics	Overall	Premenopausal	Postmenopausal	*P* value^a^
Number of patients	128	74	54	
Number of masses	144	82	62	
Age, years, Mean ± SD	41.19 ± 16.97	28.95 ± 9.51	57.96 ± 8.36	< 0.001^b^
Bilateral involvement, n(%)	16 (12.50)	8 (10.81)	8 (14.81)	0.498
Increased CA-125 levels, n(%)	45 (35.16)	14 (18.92)	31 (57.41)	< 0.001^b^
Malignant masses, n(%)	40 (27.78)	9 (10.98)	31 (50.00)	< 0.001^b^

Note: CA125, cancer antigen 125; SD, standard deviation

a: use *t*-test, chi-squared (χ2) or Fisher’s exact tests

b: P < 0.05, the difference is statistically significant

As 16 patients had bilateral adnexal lesions, the current study ultimately included 144 adnexal masses, 40(27.78%) of which were malignant and 104(72.22%)were benign. These were confirmed by postoperative pathology analysis. The most frequent benign tumour was teratoma (32/104; 30.77%), while the most common malignant tumour was serous adenocarcinoma (25/40; 62.50%). Compared with premenopausal women, postmenopausal women enrolled in the study had a much higher rate of malignant masses (50.00% vs. 10.98%, *p* < 0.001). Details of the pathology results are shown in Table 2. In the postmenopausal group, there were 8(25.81%)cases of bilateral adnexal involvement, of which 5 cases had the same pathological diagnosis of adnexal masses (3 fibromas, 1 hydrosalpinx, 1 serous cystadenocarcinoma), while 3 cases had different diagnoses (1 teratoma and fibroma, 1 metastatic tumor and simple/functional cyst, 1 serous cystadenocarcinoma and fibroma) In the premenopausal group, there were 8(10.81%)cases of bilateral adnexal involvement, of which 6 cases had the same pathological diagnosis of adnexal masses (3 cases of hydrosalpinx, 2 case of fibroma, and 1 case of serous cystadenocarcinoma), while 2 cases had different diagnoses (1 case of mucinous cystadenoma and simple/functional cyst, 1 case of fibroma and hydrosalpinx).

**Table 2:** Pathological diagnoses of the 144 adnexal masses

Histology	Overall	Premenopausal	Postmenopausal
Number of masses	144	82	62
Benign masses, n(%)	104 (72.22)	73 (89.02)	31 (50.00)
Teratoma	32 (30.77)	27 (36.99)	5 (16.13)
Simple/functional cyst	27 (25.96)	21 (28.77)	6 (19.35)
Serous cystadenoma	10 (9.62)	6 (8.22)	4 (12.90)
Mucinous cystadenoma	4 (3.85)	3 (4.11)	1 (3.22)
Ovarian endometrial cyst	5 (4.81)	4 (5.48)	1 (3.22)
Fibroma	11 (10.58)	3 (4.11)	8 (25.81)
Hydrosalpinx	8 (7.69)	5 (6.85)	3 (9.68)
Tubo-ovarian abscess	3 (2.88)	0 (0)	3 (9.68)
Other benign masses	4 (3.85)	4 (5.48)	0 (0)
Malignant masses, n(%)	40 (27.78)	9 (10.98)	31 (50.00)
Serous cystadenocarcinoma	25 (62.50)	3 (33.33)	22 (70.97)
Clear-cell carcinoma	4 (10.00)	2 (22.22)	2 (6.45)
Granulosa-cell tumor	3 (7.50)	2 (22.22)	1 (3.22)
Mucinous adenocarcinoma	2 (5.00)	0 (0)	2 (6.45)
Metastasis	3 (7.50)	0 (0)	3 (9.68)
Carcinosarcoma	1 (2.50)	1 (11.11)	0 (0)
Endometrioid carcinoma	1 (2.50)	0 (0)	1 (3.22)
Disgerminoma	1 (2.50)	1 (11.11)	0 (0)

### Comparison of diagnostic effectiveness between two scoring systems

**Fig. 1:**
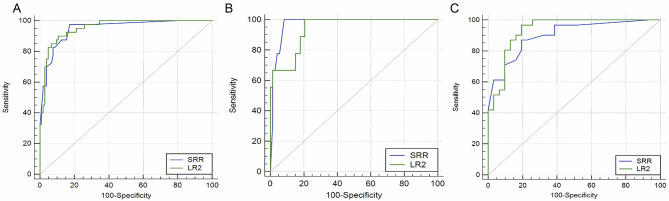
The ROC curves for performances of IOTA SRR and LR2 scoring systems in differentiating between benign and malignant adnexal masses using the data from overall patients (**A**), premenopausal women (**B**) and postmenopausal women (**C**). IOTA, International Ovarian of Tumor Analysis; SRR, Simple Rules Risk; LR2, Logistic Regression Model 2

The ROC curve of malignant adnexal masses diagnosed by physicians using the two scoring systems is shown in Fig. 1. Among all patients, the AUC of the SRR scoring system and LR2 model were 0.949 (95% CI: 0.899–0.978) and 0.955 (95% CI: 0.907–0.982), respectively. In terms of the patient population, the AUC of the SRR scoring system and LR2 model in premenopausal women were 0.974 (95% CI: 0.912–0.997) and 0.939 (95% CI: 0.864–0.980), respectively. In postmenopausal women, the AUC of the SRR scoring system and LR2 model were 0.902 (95% CI: 0.800–0.963) and 0.935 (95% CI: 0.843–0.982), respectively. There was no statistically significant difference in AUC between the two physicians using the SRR scoring system and LR2 model for diagnosis, whether in the entire population, premenopausal or postmenopausal women (*p* = 0.766, 0.371, 0.331, respectively).

**Fig. 2:**
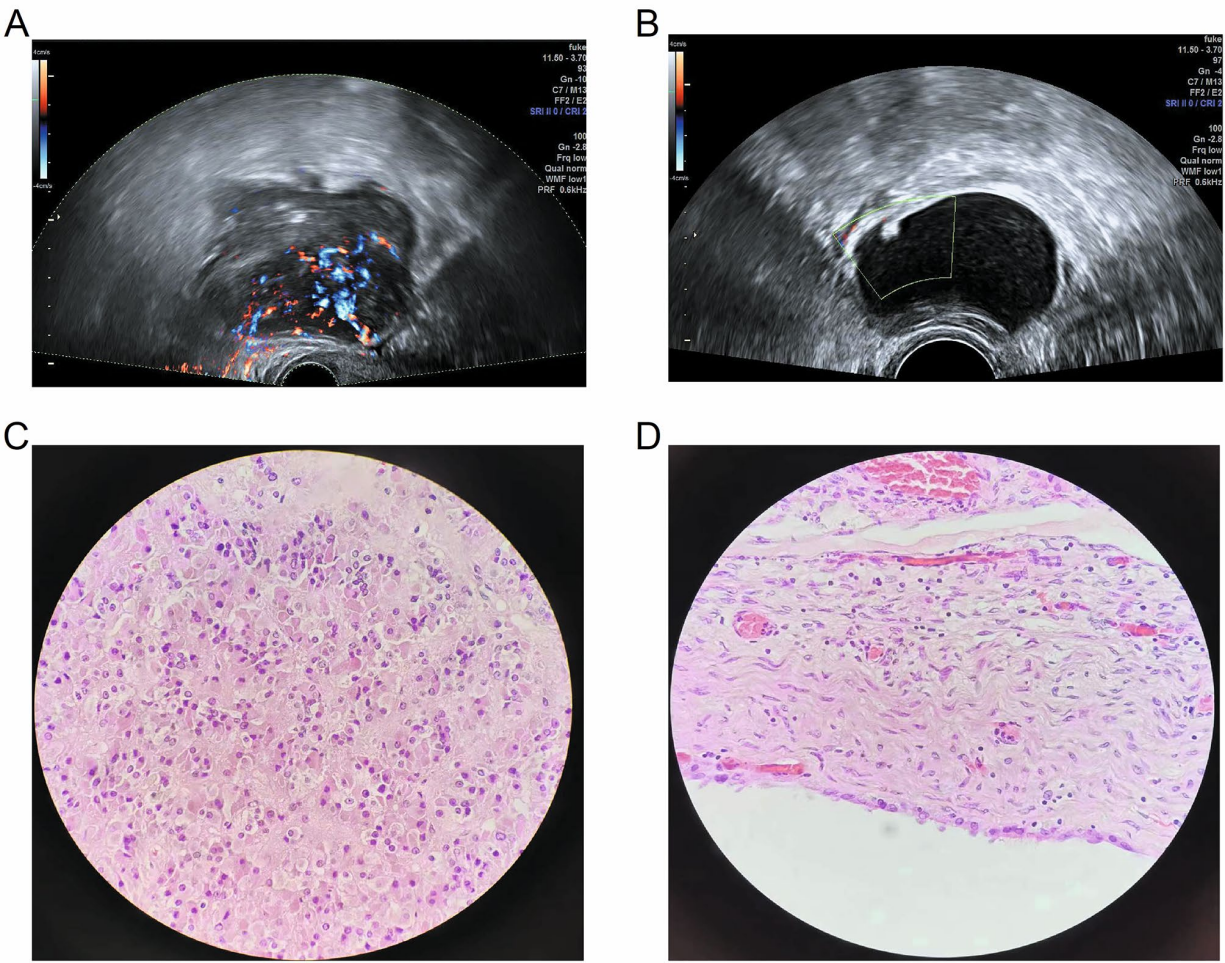
The representative ultrasound images with final histopathology diagnosis. (**A, C**) Image of a pathologically proven serous adenocarcinoma from a 45-year-old woman is shown. The mass was classified as high risk by SRR and LR2 scoring systems, respectively. (**B, D**) Image of a pathologically proven serous cystadenoma from a 51-year-old woman is shown. The mass was classified as low risk by SRR and LR2 scoring systems, respectively. SRR, Simple Rules Risk; LR2, Logistic Regression Model 2

Physicians had good consistency in the preoperative diagnosis of malignant adnexal masses using the SRR and LR2 scoring systems, with a total consistency of 84.03% and a k-value of 0.783 (95% CI: 0.70-0.864). Representative US images with final histopathology diagnoses are shown in Fig. 2.

Using the thresholds defined in the current study, 94 (65.28%) of all adnexal masses were classified as low risk (5 malignant and 89 benign) and 50 (34.72%) were classified as high risk (35 malignant and 15 benign) by the SRR scoring system. The malignancy rates for each group, according to the SRR score, were 5.32% (5/94) and 70.00% (35/50), respectively. The sensitivity was calculated as being 97.5% (95% CI: 86.8–99.9%), specificity was 82.7% (95% CI: 74.0–89.4%), positive predictive value (PPV)was 68.4% (95% CI: 58.7–76.8%), negative predictive value (NPV) was 98.9% (95% CI: 92.5–99.8%) and youden index was 0.802. See Table 3 for details.

**Table 3:** The diagnostic validity of the two scoring systems

Scoring system	AUC (95% CI)	Sensitivity (%)	Specificity (%)	PPV (%)	NPV (%)	Youden index
SRR						
Overall patients	0.949 (0.899–0.978)	97.5 (86.8–99.9)	82.7 (74.0-89.4)	68.4 (58.7–76.8)	98.9 (92.5–99.8)	0.802
Premenopausal women	0.974 (0.912–0.997)	100.0 (66.4–100.0)	91.78 (83.0-96.9)	60.0 (41.1–76.4)	100	0.918
Postmenopausal women	0.902 (0.800-0.963)	87.1 (70.2–96.4)	80.7 (62.5–92.5)	81.8 (68.4–90.3)	86.2 (71.1–94.1)	0.677
LR2						
Overall patients	0.955 (0.907–0.982)^a^	90.0 (76.5–97.2)	89.4 (81.9–94.6)	76.6 (65.0-85.2)	95.9 (90.2–98.3)	0.794
Premenopausal women	0.939 (0.864–0.980)^b^	100.0 (66.4–100.0)	79.45 (68.4–88.0)	37.5 (27.6–48.5)	100	0.795
Postmenopausal women	0.935 (0.843–0.982)^c^	96.77 (83.5–99.9)	80.7 (62.5–92.5)	83.3 (70.8–91.1)	96.2 (78.3–99.4)	0.774

Note: AUC, area under the curve; PPV, positive predictive value; NPV, negative predictive value; CI, confidence interval; SRR, Simple Rules Risk; LR2, Logistic Regression Model 2;

a: The Delong test P-value of AUC for SRR and LR2 in the overall patients is 0.766;

b: The Delong test P-value of AUC for SRR and LR2 in premenopausal women is 0.371;

c: The Delong test P-value of AUC for SRR and LR2 in postmenopausal women is 0.331

Using the LR2 scoring system to classify adnexal lesions, 97 (67.36%) masses were classified as low risk (4 malignant and 93 benign) and 47 (32.64%) were classified as high risk (36 malignant and 11 benign). The malignancy rates for each group, according to the LR2 model, were 4.12% (4/97) and 76.60% (36/47), respectively. The sensitivity was calculated as being 90.0% (95% CI: 76.5–97.2%), specificity was 89.4% (95% CI: 81.9–94.6%), PPV was 76.6% (95% CI: 65.0–85.2%), NPV was 95.9% (95% CI: 90.2–98.3%) and youden index was 0.794. See Table 3 for details.

## Discussion

The current study aimed to compare the diagnostic accuracy of two US-based predictive scoring systems, SRR and LR2 (both proposed by the IOTA group), for discriminating between malignant and benign adnexal masses in Chinese patients. The current study’s results showed that both scoring systems exhibited high diagnostic accuracy when performed by junior doctors. The two scoring systems had good agreement in the diagnosis of malignant adnexal tumours, and the diagnostic performance of the two systems was similar.

The IOTA LR2 scoring system is one of the best-performing US-based adnexal lesion diagnostic systems currently in use in clinical practice [12]. Sensitivity and specificity are comprehensive indicators for evaluating diagnostic tests, as they indicate the ability to accurately identify the proportion of benign and malignant adnexal masses. In clinical decision-making, high-sensitivity models help to rule out disease and reduce the risk of missed diagnosis, and high-specificity models help to rule out non-disease states and reduce the risk of overdiagnosisv [21].The balance of sensitivity and specificity is an important factor to consider in clinical decision-making, and high sensitivity is considered to be preferred in screening because it is crucial to ensure fewer missed diagnoses; whereas in confirmatory testing, because reducing misdiagnosis is key, specificity is considered a priority [23]. The results of the current study showed that the sensitivity of the LR2 scoring system in distinguishing the nature of adnexal masses was 90.0% (95% CI: 76.5–97.2), and the specificity was 89.4% (95% CI: 81.9–94.6), with slightly higher sensitivity than specificity. The sensitivity and specificity of the research results are higher compared with the result presented by Zhao et al. [17], where the diagnostic effect of LR2 for distinguishing malignant adnexal masses was equivalent to that of experienced physicians, with a sensitivity of 79.6% and a specificity of 88.1%. However, compared with the model sensitivity (97.0%) obtained from the external validation study of LR2 conducted by Nunes et al. [24], the sensitivity obtained in the current study was lower. Overall, the LR2 scoring system has high sensitivity and specificity for distinguishing benign and malignant tumours in patients with adnexal masses. Shimada et al. [8] also noted that in distinguishing between benign and malignant tumours, the sensitivity of the LR2 system was comparable to that of MRI, and its specificity was higher than that of MRI.

The IOTA SRR scoring system was developed to estimate the risk of malignancy based on the B- and M-features of the SR model and centre type [20]. The IOTA proposes 10 simple criteria (5 benign B features and 5 malignant M features) and represents a large sample study with diagnostic efficacy verified in multiple centres in different countries [25, 26]; however, there is a lack of research validation involving its use in China. The results of the current study showed that the AUC of the SRR scoring system was 0.949 (95% CI: 0.899–0.978), the sensitivity was 97.5% (95% CI: 86.8–99.9) and the specificity was 82.7% (95% CI: 74.0–89.4), with sensitivity higher than specificity. The AUC of the SRRS scoring system cabinet obtained from the current study was higher than that presented by Hiett et al. (AUC: 0.941) [13]. Wynants et al. [27] reported that SRR was clinically more useful for identifying patients with adnexal masses than other models, such as RMI and ROMA. Overall, the diagnostic performance of the SRR scoring system was robust and universally applicable. Multiple studies have shown that the SRR scoring system significantly improved the diagnostic performance and sensitivity of non-professional US technicians [28, 29].

The comparability of the diagnostic performance of the IOTA LR2 and SR systems has previously been established. Niemi et al. [30] evaluated the performance of different models for predicting malignant or benign pelvic masses in postmenopausal women and found that the SR and LR2 models had similar sensitivity and specificity [30]. Furthermore, a meta-analysis based on data from 47 articles (19,674 adnexal tumours) showed that the LR2 system had a diagnostic accuracy similar to the SR model in terms of differentiating between benign and malignant adnexal masses prior to surgery [15]. The results of the current study showed that both models had high sensitivity and specificity, with sensitivity higher than specificity, indicating that they had good effects in both prediction and confirmation in clinical practice, with predictive potential being superior to confirmatory potential. For inexperienced junior physicians, improving the accuracy of ultrasound diagnosis is a key issue. Our results indicated that both SRR and LR2 systems had high diagnostic efficiency and could assist junior physicians in diagnosing ovarian cancer patients with ultrasound results. However, the SR model has several shortcomings, including inconclusive results in a percentage of cases and the absence of a malignancy risk estimation. The SRR scoring system was developed to overcome the shortcomings of the SR model, but no previous studies have been published to compare the diagnostic performance of the SRR and LR2 systems for predicting malignant adnexal masses. The present study directly compared the diagnostic performance of these two scoring systems in Chinese patients and found that the AUC, sensitivity and specificity of the SRR and LR2 scoring systems, when performed by junior physicians, were comparable. In addition, the two systems showed good agreement in terms of diagnosing malignant adnexal tumours, with 84.03% total agreement and a k-value of 0.783, indicating that the diagnostic performance of the two systems is similar. In predicting malignant adnexal tumors, the sensitivity of the SRR system was higher than that of the LR2 system, but the specificity was lower than that of the LR2 system. However, the difference between the two was not statistically significant, and SRR was not superior to LR2. Both systems seem to be able to compensate to some extent for the lack of experience of junior physicians.

The present study has several limitations, the most noteworthy of which was its low sample size. Previous studies with a similar aim showed that a sample size of 100 was sufficient for validating the study objective [12, 29]. Although our sample size was above 100, it would be worthwhile to substantiate the results with a larger sample. Due to the small sample size, the studied samples may not fully represent the characteristics and diversity of the target population and may not be able to capture the true size of the effects, thereby reducing the ability to detect true effects and limiting the external validity of the research results Additionally, because of its retrospective nature, the study could not obtain dynamic images to sufficiently evaluate each adnexal mass. This may have caused misjudgements of certain US features. Large sample studies may reduce the impact of individual abnormal observations, resulting in more stable results that are more representative. At the same time, research results are more likely to have statistical significance, with smaller estimated standard errors, increasing the credibility of the study. In addition, the data of the current study was collected in a single center, and there may be regional differences in the selection of research subjects, resulting in selection bias and possibly limiting the widespread inference of research results. Finally, the current study did not do a sensitivity analysis and only used criteria commonly used in previous studies to determine whether the findings were benign or malignant, which may limit the external validity of the findings.

## Conclusion

In conclusion, the IOTA SRR and IOTA LR2 scoring systems could effectively distinguish benign from malignant adnexal masses. Both scoring systems had high sensitivity and specificity, with SRR system having higher sensitivity and lower specificity than LR2 system. The two systems have high diagnostic efficiency and can serve as important reference for clinical decision-making. They can also serve as a powerful tool to help junior physicians improve their diagnostic ability of malignant adnexal masses and provide patients with earlier intervention and treatment. This will have an important impact on improving the diagnostic level of adnexal tumours and the prognosis of patients in clinical practice. However, it should be noted that the current study is a single center study with a small sample size, and the results may have certain limitations. In future research, we will collect more comprehensive data, conduct prospective studies in multiple centers, and conduct sensitivity analysis to further investigate the diagnostic efficacy of the two systems in clinical practice, and explore the mechanisms and influencing factors of the two systems in assisting junior physicians in diagnosis.

## Data Availability

All data generated or analyzed during the current study are included in this published article.
